# From Data to Wisdom: Biomedical Knowledge Graphs for Real-World Data Insights

**DOI:** 10.1007/s10916-023-01951-2

**Published:** 2023-05-17

**Authors:** Katrin Hänsel, Sarah N. Dudgeon, Kei-Hoi Cheung, Thomas J. S. Durant, Wade L. Schulz

**Affiliations:** 1 Department of Laboratory Medicine, Yale School of Medicine, New Haven, CT USA; 2 Section of Biomedical Informatics, Department of Emergency Medicine, Yale School of Medicine, 55 Park Street, PS 210, New Haven, CT 06510 USA; 3 Department of Biostatistics, Yale School of Public Health, New Haven, CT USA

**Keywords:** Biomedical knowledge graph, Medical knowledge curation, Healthcare applications, Clinical outcome prediction

## Abstract

Graph data models are an emerging approach to structure clinical and biomedical information. These models offer intriguing opportunities for novel approaches in healthcare, such as disease phenotyping, risk prediction, and personalized precision care. The combination of data and information in a graph model to create knowledge graphs has rapidly expanded in biomedical research, but the integration of real-world data from the electronic health record has been limited. To broadly apply knowledge graphs to EHR and other real-world data, a deeper understanding of how to represent these data in a standardized graph model is needed. We provide an overview of the state-of-the-art research for clinical and biomedical data integration and summarize the potential to accelerate healthcare and precision medicine research through insight generation from integrated knowledge graphs.

## Introduction

The term Knowledge Graph (KG) was originally coined by Google [1] as a concept for structuring information into graphs to enhance web search. KGs are now ubiquitous around us and they power the modern web from tailored search results to personalized recommendations. KGs have also gained traction in biomedicine to represent public biomedical knowledge, integrate immunological research data, and advance drug discovery [2] and in healthcare, KGs have been used to support applications such as clinical decision support systems [3]. However, the use of KGs to model and drive discovery from real-world data (RWD), such as data from the electronic health record (EHR), has been limited. Among the emerging literature, findings suggest that there is a likely benefit from augmenting healthcare data with external knowledge, such as biomedical KGs, for applications such as disease risk prediction [4]. In this manuscript, we provide a brief overview of KGs and describe recent successes and future applications for healthcare data.

## Technical background: Graphs to knowledge graphs

**Fig. 1:**
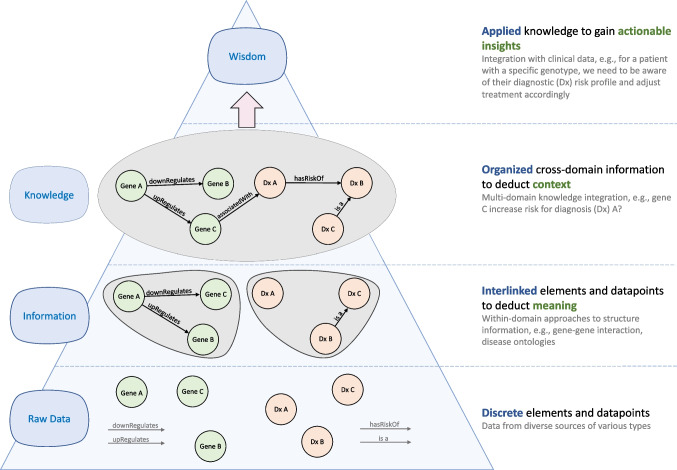
Application of the Data-to-Wisdom Pyramid to biomedical graph data. Individual data elements can be connected within a graph to model information. The cross-domain aggregation of this information embeds knowledge directly within the data model, resulting in a knowledge graph (KG). These graphs can be used to generate insights or wisdom, such as clinical decision support or knowledge-enabled explanations, compared to a data model that may lack the more detailed context and relationships that are present in a graph model.

Biomedical data have an inherent graph structure, such as drug-disease interactions that capture data from multiple domains represented as bipartite networks, or protein-protein interactomes which can be represented in a unipartite network [5, 6]. However, traditional approaches to model and analyze these data are often reductionist, relying on constrained, tabular models without machine-readable context. Graphs provide the opportunity to model not just data, but also metadata and complex relationships between data elements. As opposed to the rows and columns used with more traditional approaches, graph-oriented models are represented with nodes, or vertices, and edges, or relationships. When data and information are joined in such a structure, the resulting data representation is referred to as a *knowledge graph*, which provides a computationally accessible (i.e., machine-readable) representation of relationships between disparate biologic systems information. From these KGs, novel relationships can be identified and used to generate wisdom and actionable insights, as demonstrated in recent publications from the fields of computational biology and the life sciences [7-9]. In Fig. 1, we illustrate the application of the data-to-wisdom pyramid [10] to graph structures, which demonstrates how stepwise structuring, contextualization, and integration of graph-oriented data, information, knowledge, and wisdom can be used to drive insight generation.

## Knowledge graph applications

The use of graph models and KGs has increased with access to more accessible graph database software. These technologies have shown efficient query performance, offer unique visualization tools, and have integrated specialized analytics packages for data science applications [11].

In recent years, biomedical researchers have increasingly adopted these technologies to better model the complexity of biological systems. In 2019, Bukhari et al. [12] used Neo4J – an enterprise graph database – to build a KG from multiple, variably formatted, publicly available data sources to support systems-based vaccinology. Related research demonstrates further insights derived from novel KG analysis [12]. For example, Youn et al. [9] describe the construction of an *Escherichia coli* antibiotic resistance KG that integrates 10 publicly available data sources. With this approach, the authors identified six novel *Escherichia coli* resistance genes that were identified via graph-based *in-silico* link predictions which were then validated biologically.

Other work has demonstrated the reduction of complex, graph-structured information and knowledge into simpler mathematical representations, termed embeddings, which can be used to retain the structural information encoded in graphs to facilitate downstream processing and analytics [13]. Embeddings build the basis for many graph-based data science tasks, such as prediction of edges between nodes, classification of node types, or clustering of related nodes [14]. Embeddings of large biomedical KGs based on the Unified Medical Language System (UMLS) have been used to predict similarity in meaning of medical concepts, a method that can be applied for natural language processing (NLP) of clinical texts [15].

## Knowledge graph applications in healthcare

Graph networks have rapidly gained traction in basic and translational data sets and there is high potential for graph databases to be applied in healthcare [16]. However, less has been published on the use of graph representations to model patient data as shown in a recent systematic review by Schrodt et al. [3], which only identified 11 articles that used graphs to represent clinical data, such as laboratory results and comorbidities (Table 1).

**Table 1:** Overview of biomedical and clinical use cases that can be addressed using graph and knowledge graph-based approaches

**Healthcare Use Case**	**Graph and Knowledge Graph Mechanics: Explanation and examples**
**Drug repurposing,** **Comorbid risk prediction**	**Link prediction:** Prediction of the likelihood of an existing edge between two nodes based on the entirety of the knowledge. For example, prediction of edge likelihood of drug compound and patient to predict personal risk of adverse reaction or links between two diseases posing a high comorbidity risk [7, 17].
**Disease subtyping**	**Community detection/graph clustering:** Identification of highly connected regions within a real-world data graph that can identify patients with a high similarity, e.g., patients with a certain disease subtype [8].
**Outcome, status, and risk prediction**	**Node classification:** Prediction of the likelihood of a patient node being assigned a label based on the entirety of their medical data. For example, patient node gets assigned a disease risk label [4].
**Visual insights**	**Graph layout and visualization:** There have been several studies into the visualization and lay outing of graph-structured data, e.g., biomedical or healthcare data, for aiding human interpretability and pattern recognition [18].
**Complex patient data queries**	**Graph traversal:** The inherent connected representation in graphs allows for the easy traversal of the graph to identify pieces of information that are separated by several nodes. When combining patient data with terminological knowledge, this allows for complex queries, e.g., identification of all patients based on a medical condition and its subtypes [19].

The longitudinal efforts of Baranzini and colleagues have demonstrated how biomedical KGs, such as Hetionet [5] and its successor, the Scalable Precision Medicine Open Knowledge Engine (SPOKE), can be used to integrate information such as biological processes, molecular functions, complex diseases, as well as macro-cellular structures, proteins, and pathways for use in a variety of biomedical and healthcare applications. In 2019, Nelson et al. [20] described the connection of SPOKE with clinical electronic health record (EHR) data by leveraging shared concepts between the KG and EHR. Compared to using EHR data alone, the enrichment of clinical data with KG embeddings from SPOKE improved the performance of a downstream machine learning-based algorithm to predict the future diagnosis of multiple sclerosis [4]. It was hypothesized that integrating the KG data compensated for missing and/or incomplete data in the EHR. While this study showed promising results in the detection of the prodromal phase of multiple sclerosis, the generalizability of their embedding approach for other EHR data sets and predictive tasks remains unknown.

## Limitations to knowledge graph implementation

While KGs are an intriguing solution to layer biomedical knowledge on clinical data sets, there remain challenges related to implementation and generalizable application. Firstly, biomedicine is an ever-evolving field with over one million new publications added to PubMed each year – nearly one publication per minute [21]. This poses the challenge of how to efficiently and accurately integrate this newly generated knowledge into a graph model. Secondly, the selection of appropriate node types and vocabularies to model data from diverse data sources can be a time-consuming and imperfect process, but one that is necessary to connect typically disparate data sets. Thirdly, while much has been done to create relational common data models for real-world data, no such standards exist today for graph-based data models. There is a need to integrate knowledge graphs with existing ontologies and linked data that have been implemented using semantic web technologies such as the Resource Description Framework (RDF). Finally, additional studies assessing the performance of graph-based clinical machine learning and artificial intelligence are needed to demonstrate the usefulness of knowledge graph models for these applications.

## Conclusions

The integration of EHR data into KGs represents a promising approach to enhance clinical and translational research. However, these efforts are still in the early stages of development and require more in-depth testing and translation before they will be routinely placed into practice. The enrichment of EHR and other real-world data with broad biomedical knowledge bases is a lofty, but intriguing and alluring goal. As a scientific community, through the accumulation and contextualization of vast amounts of information and knowledge, we have the opportunity to create next-generation data models that can embed knowledge to provide greater context for analytics and machine learning applications, drive applications that provide actionable insights, and advance the field of real-world evidence generation. But to make these data models accessible and generalizable, further research is needed to understand best practices regarding how clinical data can be transformed into graph models to support downstream analytic tasks. Achieving this will allow us to better model complex, multi-modal information borne out from discoveries of the past and years to come.

## Data Availability

As a comment article, this manuscript did not analyze any specific data sets.
